# Improved image quality of low‐dose thoracic CT examinations with a new postprocessing software†

**DOI:** 10.1120/jacmp.v11i3.3242

**Published:** 2010-05-25

**Authors:** Anne Catrine Traegde Martinsen, Hilde Kjernlie Saether, Dag Rune Olsen, Per Aage Wolff, Per Skaane

**Affiliations:** ^1^ The Interventional Centre Oslo University Hospital Oslo Norway; ^2^ Institute of Hospital Medicine University of Oslo Norway; ^3^ Department of Radiology Oslo University Hospital Oslo Norway; ^4^ Institute of Cancer Research Oslo University Hospital Oslo Norway; ^5^ Department of Physics University of Oslo Oslo Norway

**Keywords:** CT, low‐dose CT, chest CT, postprocessing filter

## Abstract

In 2008 a phantom study indicated that there is a potential for reducing the CT doses when using a new postprocessing filter. The purpose of this study was to test this new postprocessing filter clinically for low‐dose chest CT examinations, to assess whether the diagnostic performance is the same or improved. A standardized clinical chest CT protocol was used on patients with colorectal cancer. Only mA settings changed between patients according to patient size. One standard and one low‐dose chest protocol were performed for all patients. The low‐dose images were postprocessed with a new software filter, which provides context‐controlled restoration of digital images by using adaptive filters. Three radiologists assessed randomly all the images independently. A total of 24 scan series were evaluated with respect to image quality according to quality criteria from the European guidelines for chest CT using a five‐point scale; 576 details were assessed. Overall mean score is the average score for all details rated for all three readers for all full‐dose series, low‐dose series and low‐dose enhanced series, respectively. The statistical methods used for comparison were paired sampled t‐test and intraclass correlation coefficient. The postprocessing filter improved the diagnostic performance compared to the unenhanced low‐dose images. Mean score for full‐dose, low‐dose and low‐dose enhanced series were 3.8, 3.0 and 3.3, respectively. For all patients the full‐dose series gave higher scores than the low‐dose series. Intraclass correlation coefficients were 0.2, 0.1 and 0.3 for the full‐dose, low‐dose and low‐dose enhanced series, respectively. There is a potential for improving diagnostic performance of low‐dose CT chest examinations using this new postprocessing filter.

PACS number: 87.57.C‐, 87.57.Q‐

## I. INTRODUCTION

Fourteen percent of total worldwide exposure to radiation is from diagnostic X‐ray exposure.^(^ ^1^ ^)^ In European and US hospitals, the CT examinations account for more than 50% of the collective effective dose^(^ ^2^ ^–^ ^4^ ^)^ associated with medical exposure.

For doses of 100 mGy and higher, there is a proven risk for radiation‐related cancer induction, and there is no rational for assuming a low‐dose threshold for cancer induction.^(^ ^5^ ^)^ In radiation protection it is, therefore, a general assumption that the risk for stochastic effects increases linearly with dose, without any threshold.

A British study indicates that about 0.6% of the cumulative risk of cancer in the UK could be attributable to diagnostic X‐ray, equivalent to 700 cases.^(^ ^1^ ^)^ In the study, the estimated number of radiation‐induced cases of lung cancers per year based on 1998 UK population was 61 for both sexes combined. Mayo et al.^(^ ^6^ ^)^ concludes that the complex relationship between radiation exposure, image noise and diagnostic accuracy should be investigated further to establish the minimum radiation dose required to provide adequate diagnostic image quality. The main goal in optimizing CT examinations is to reduce the radiation and at the same time maintain or even improve diagnostic accuracy.

In order to improve diagnostic image quality with respect to noise supression, low‐contrast detectability and spatial resolution without, at the same time, increasing the radiation doses from CT examinations, some manufacturers have developed stand‐alone postprocessing tools that are compatible with all commercially available CT scanners. A phantom study of CT examinations of liver lesions published in 2008, indicated a potential for reducing the doses by 30% and, at the same time, maintaining the diagnostic image quality by using a post processing filter called SharpView CT.^(^ ^7^ ^)^ This filter is applied as a postprocessing step between the scanner and the picture archive (PACS) after the image reconstruction on the CT scanner is finished, working on the processed image data not the raw data, from the scanner. The filter is intended for enhancing edges and lines, 2D adaptive noise suppression and artefacts, in addition to spatial consistency.

The aim of our study was to evaluate the postprocessing filter SharpView CT clinically for low‐dose thoracic CT examinations, and to assess if the diagnostic performance is affected using this new filter. It was not expected that the image quality for the low‐dose thoracic CT examinations would be at the same diagnostic level as the full‐dose images; rather we wanted to assess if the diagnostic performance at the low‐dose level was affected using this new filter, since low‐dose thoracic CT exams are used more and more worldwide.

## II. MATERIALS AND METHODS

A total of 13 colorectal cancer patients with suspected or already known thoracic metastasis were scanned. For five of the patients, the scan series did not cover all the chest area of interest in this study and they were consequently excluded from the study. All patients underwent standard CT scanning of liver and chest, as part of the national cancer follow‐up regime. Eight patients were included in the data analysis; four male and four female, ranging in age from 50 to 75 years; mean age was 67.6 years.

The oncologists responsible for the treatment of each patient were informed about the study. All patients were included in the study upon informed consent. Each patient underwent one full‐dose thoracic CT exam and one low‐dose thoracic CT exam.

All CT protocols in the hospital had been optimized beforehand in order to minimize the dose levels while maintaining adequate diagnostic performance.

In this study, all scans were performed using a 64‐slice CT scanner (Philips Brilliance 64, Best, The Netherlands). The scan parameters for the full‐dose thoracic CT protocol were 120 kV, 0.7s/rotation, pitch 0.9, 64 by 0.625 mm collimation and 200 mAs. In the hospital use of the automatic current selection (ACS) and dose modulation in Z‐direction (Z‐DOM) are standard. Mean pitch corrected values of weighted CT dose index (${\text{CTDI}}_{\text{vol}}$) was 8 mGy for the patients included in the study. In comparison, the national reference level for chest CT in Norway is ${\text{CTDI}}_{\text{vol}}=20\;\text{mGy}$.^(^ ^8^ ^)^

The low‐dose protocol applied in the study is based on the following parameters: 120 kV, 0.7s/rotation, pitch 0.9, collimation 64 by 0.625 mm and 30 mAs. ACS and Z‐DOM were not used for the low‐dose protocol. ${\text{CTDI}}_{\text{vol}}$ for the low‐dose scan was 1.8 mGy. The exposure level of the low‐dose protocol used in the study is comparable to dose levels used for assessing pulmonary nodules, as described in the literature.^(^ ^5^ ^,^ ^9^ ^–^ ^16^ ^)^

The reconstructed images were 2 mm thick. Two physicists and a radiographer, who were not involved in the image evaluation process, performed all imaging and the postprocessing image reconstruction.

**Figure 1:**
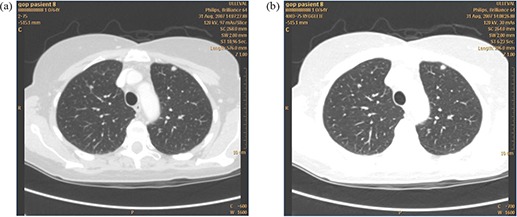
A full‐dose chest CT image (a) in which only standard reconstruction was used; a low dose chest CT image (b)for the same patient in which SharpView postprocessing filter was used.

**Figure 2:**
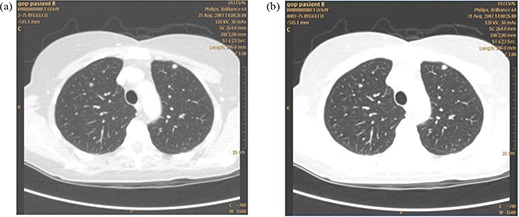
Two low‐dose chest CT images, at the same dose level, at the same position for the same patient: (a) standard reconstruction was used; (b) SharpView postprocessing filter was used.

One full‐dose and one low‐dose thoracic CT scans were performed for all patients. The low‐dose images were postprocessed using an adaptive filter named SharpView CT (SharpView AB, Linköping, Sweden). Figure 1 shows a full‐dose chest image without SharpView postprocessing and a low‐dose image with SharpView post‐processing. Figure 2 shows two low‐dose CT images with standard and with SharpView postprocessing. The image contrast is different for the full‐dose and the low‐dose images due to different radiation doses. During image assessing, the window width and window level were changed by the observers as the observers normally do in a clinical situation.

Intravenous iodine contrast was given for the full‐dose scans for all patients. The image quality criteria evaluated in this study are not influenced by the use of iodine contrast enhancement.

Three observers, all cross‐sectional radiologists, assessed the images independently. The observers were given a set of 24 scan series in total obtained at two different dose levels, 200 mAs and 30 mAs. Both standard and SharpView postprocessing were evaluated at low dose. Only the standard postprocessing was evaluated for full‐dose images.

Twenty‐four scan series and eight image quality criteria were evaluated by the three observers; in total 576 details were assessed. All series were assessed with respect to eight CT image quality criteria from the European guidelines on quality criteria for CT on a five‐point scale.^(^ ^17^ ^)^ The criteria were chosen from the list of general chest and HRCT chest criteria. Criteria for the mediastinal region, lung parenchyma and the lung tissue were chosen to assess the new postprocessing filter. Table 1 lists the criteria assessed in the study. ${\text{CTDI}}_{w}$ recommended in the guidelines are 35 and 30 mGy for the HRCT and the routine CT exam, respectively. Also the pitch corrected values of weighted CT dose index (${\text{CTDI}}_{\text{vol}}$) would be 35 and 30 mGy respectively, which is higher than both the national reference value and the doses used in this study. In total, 505 out of 576 quality criteria details were possible to rate; the remaining were not rated due to too‐short scan regions in some examinations. For some patients, certain details could consequently not be rated for all dose levels. The 505 details were included to form the basis for the statistical analyses in this study.

**Table 1:** Image quality criteria.

*The image quality criteria from European guidelines for chest CT used in this study. The observers gave a score on a scale from 1 to 5, where score* 1=not *visible; score* 2=poor/hardly *visible; score* 3=visible; *score* 4=clearly *visible; score* 5=visually *sharp.*
1: Reproduction of pulmonary fissures
2: Reproduction of small pulmonary vessels within 1 cm off the pleura
3: Reproduction of bronchial walls within 3 cm from the chest wall
4: Reproduction of major mediastinal vessels
5: Reproduction of endotracheal and endobronchial margins
6: Reproduction of trachea and central bronchial wall
7: Reproduction of lateral pleural margins
8: Reproduction of pleuromediastinal margin

Before the start of image assessment, all image quality criteria and the score scheme were explained in detail. Also the use of the quality criteria was demonstrated using sample images.

The observers were blinded to technical factors and imaging mode. To prevent learning bias, all images were randomly displayed on a review workstation. The reading process was performed in three sessions. Each reading session consisted of one scan series for each patient, randomized with respect to dose levels, such that a reading session consisted of full‐dose series for some patients, low‐dose series for some patients and low‐dose SharpView enhanced series for some patients. Image reading included three reading sessions over 16 weeks for each reader, to avoid recognition of the pathology appearing in the images.

None of the observers were involved in the image acquisition process. All images were displayed on the same review workstation. No time constraints were given.

The SharpView CT postprocessing filter identifies image features at different abstraction levels using a hierarchical approach.^(^ ^18^ ^)^ The filter combines 2D adaptive noise suppression, edge enhancement and spatial resolution.

Different acquisition settings, such as dose and reconstruction algorithm, greatly affect the image data characteristics and might, therefore, require particular filter parameters. The parameters may also be adjusted to account for anatomical variations. The enhancement is performed in different intensity value ranges, corresponding to tissue type‐specific Hounsfield Units (HU).^(^ ^19^ ^)^ The parameters can thus be set differently for different intensity ranges (tissues), making simultaneous filtering of soft and lung tissue possible in chest examination. The SharpView CT postprocessing filter was adjusted with respect to CT scanner, anatomical region of interest and dose levels to fit the thoracic CT images as perfectly as possible. Three different versions of postprocessing filter were tested before the study started, to ensure that the most optimal filter was used in the study.

The SharpView CT vendor had no role in study design, data collection, data analysis, data interpretation or in writing this paper, except the sentences describing the functionality of the Sharpview algorithm.

A paired sampled t‐test with a 95% level of confidence was used to compare diagnostic performance of images with and without postprocessing image enhancement. Inter‐observer differences were assessed by using intraclass correlation coefficient (ICC) test with 95% confidence interval. ICC ranges from 1 to 0: ICC=1 corresponds to complete agreement between the observers, and ICC=0 corresponds to no agreement at all between the observers.

The overall mean scores is the mean score for all details rated for all readers for all full‐dose series, low‐dose series and low‐dose enhanced series, respectively.

## III. RESULTS

**Figure 3:**
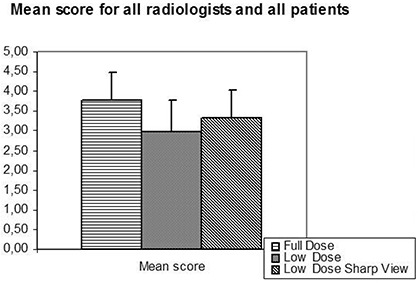
Overall mean score (the average score for all details rated for all full‐dose series, low‐dose series and low‐dose enhanced series) for all three readers. The full‐dose series gave a significantly higher score than both the low‐dose series and the SharpView enhanced low‐dose series. The SharpView enhanced low‐dose series gave a significantly higher score than the low‐dose series.

The overall mean scores for all image quality details for all full‐dose series, all low‐dose series and all low‐dose enhanced series for all three readers were 3.8, 3.0 and 3.3, respectively. The overall image quality scores for all examinations and observers was significantly higher for the full‐dose series compared to both the low‐dose series and enhanced low‐dose series (p<0.05) (Fig. 3). The overall mean scores for the SharpView enhanced low‐dose series was higher than the overall mean scores for the low‐dose series (3.3 versus 3.0, respectively, p<0.05).

**Figure 4:**
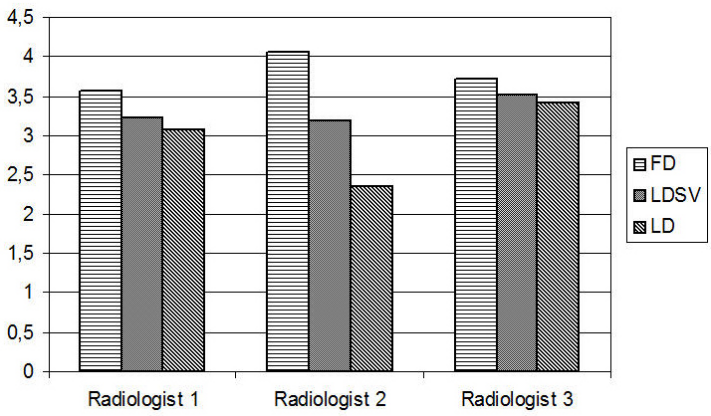
Variation in overall score between radiologists. For all radiologists the total score is highest for the full‐dose (FD) images and lowest for the low‐dose (LD) images. SV is the SharpView postprocessed images; FD is the mean score for each radiologist of the full‐dose series; LD is the mean score for each radiologist of the low‐dose series.

The three observers consistently reported overall image quality of the full‐dose series superior to that of both the low‐dose series and the low‐dose series with SharpView image quality enhancement (Fig. 4). The scores for the full‐dose, low‐dose and the low‐dose SharpView postprocessed series were 3.6, 3.1 and 3.3 for reader one, 4.1, 2.4, and 3.2 for reader two, and 3.7, 3.4 and 3.5 for reader three. The difference in image quality score between reader 1 and 2 for the full‐dose and the low‐dose SharpView postprocessed series was not significant. For observer 3, the image quality scores were significantly higher for the full dose. For all observers, the SharpView postprocessed series gave a significantly higher image quality score than the low‐dose standard (Fig. 4).

**Figure 5:**
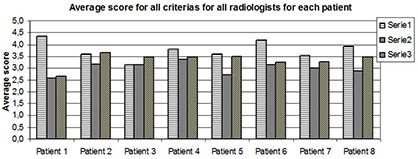
The mean score for all criteria for all radiologists for each patient. For all patients, the full‐dose series gave a higher score than the other two series (except for patient 2 and 3), and the low‐dose SharpView enhanced series gave higher score than the low‐dose series.

For each individual patient (except patient 2 and 3), the full‐dose series had the highest mean scores. A paired t‐test showed that the difference between full‐dose and SharpView enhanced low‐dose series is not statistically significant (Fig. 5) for five of the patients. The difference in score between full‐dose and low‐dose series is significant for all patients, except one (p<0.05). The SharpView enhanced low‐dose series gave a higher score than the low‐dose series for all patients.

**Figure 6:**
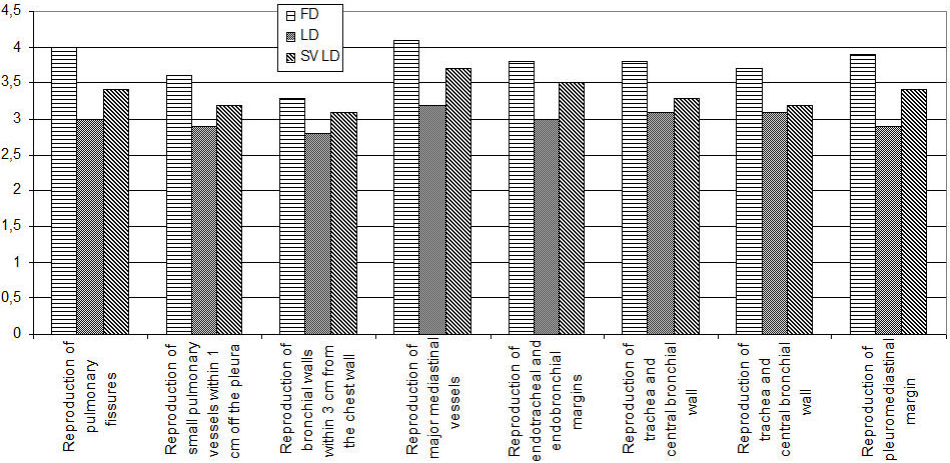
The mean score for all radiologists and patients for each quality criteria. For all criteria, the full‐dose series gave the significantly highest score (except for patient 2 and 3), and the low‐dose SharpView enhanced series gave higher score than the low‐dose series. The difference between the SharpView enhanced series and the low‐dose series is not significant.

The full‐dose series scored significantly higher than the other series for each image quality criteria. The scores for each image quality criteria ranged between 3.3 and 4.1 for the full‐dose series, between 2.8 and 3.3 for the low‐dose series, and ranged between 3.1 and 3.5 for the low‐dose SharpView postprocessed series. SharpView postprocessed low‐dose series had a higher score than the low‐dose series for all image quality criteria. The difference in scores were significant for criterion 2: “Reproduction of small pulmonary vessels within 1 cm off the pleura” (score 3.2 versus 2.9 for the low‐dose SharpView enhanced and the low‐dose series, respectively, p<0.05), criterion 4: “Reproduction of major mediastinal vessels” (score 3.7 versus 3.2 for the low‐dose SharpView enhanced and the low‐dose series, respectively, p<0.05), criterion 5: “Reproduction of endotracheal and endobronchial margins” (score 3.5 versus 3.0 for the low‐dose Sharpview enhanced and the low dose series respectively, p<0.05) and criterion 8 “Reproduction of pleuromediastinal margin” (score 3.4 versus 2.9 for the low dose SharpView enhanced and the low‐dose series, respectively, p<0.05). (See Table 1 and Fig. 6.) The difference was not significant for the criteria: “Reproduction of pulmonary fissures”, “Reproduction of bronchial walls within 3 cm from the chest wall”, “Reproduction of trachea and central bronchial wall” and “Reproduction of lateral pleural margins”.

**Figure 7:**
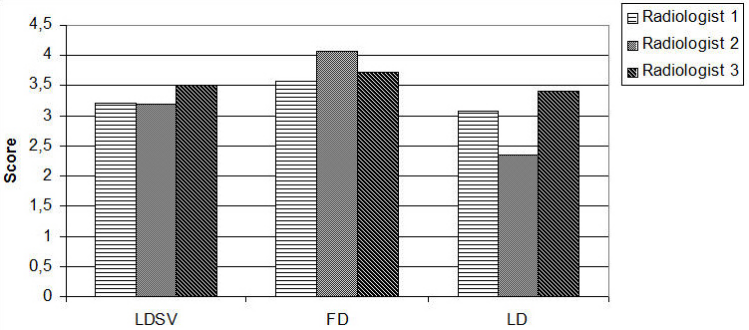
The interobserver differences for the different dose levels. LDSV is the mean score for each radiologist of the low‐dose SharpView enhanced series; FD is the mean score for each radiologist of the full‐dose series; LD is the mean score for each radiologist of the low‐dose series.

The mean score for all patients and all image quality criteria for each radiologist were above 3 for both the full‐dose and low‐dose SharpView enhanced series. The mean scores for all patients and all image quality criteria for the full‐dose series were 3.6, 4.1 and 3.1 for reader 1, 2 and 3, respectively. The mean score for low‐dose series were 3.1, 2.4 and 3.4 and the mean scores for low‐dose SharpView enhanced series were 3.3, 3.2 and 3.5 for reader 1, 2 and 3, respectively (Fig. 7). For the low‐dose series, the mean scores ranged between 2.4 and 3.4. The full‐dose series had the highest scores for all observers. Observer 3 had higher scores than the other observers for both the low‐dose series and the low‐dose SharpView postprocessed series. (Observer 3 is a specialist in chest CT.) SharpView postprocessing resulted in the best agreement between the observers. Intraclass correlation coefficients (ICC) were 0.2 for full‐dose images, 0.1 for low‐dose images and 0.3 for SharpView low‐dose images.

## IV. DISCUSSION

A previous study on the possible diagnostic performance of SharpView using a liver phantom demonstrated that the image quality was the same or even improved at 30% reduction in dose when using SharpView.^(^ ^7^ ^)^ In this study, SharpView was tested clinically at low‐dose levels and compared to full‐dose images without SharpView postprocessing.

The low‐dose series always had a lower score than the SharpView enhanced series, indicating that the use of postprocessing filters like SharpView may improve the diagnostic image quality significantly for thoracic CT exams.

The difference in average score between low‐dose and full‐dose series for all observers, patients and image quality criteria is 0.8 (score 3.8 for full‐dose and 3.0 for low‐dose) (Fig. 3). This difference in diagnostic performance has to be evaluated against the fact that full‐dose series gives six times the dose of the low‐dose series. The modest increase in image quality using the full‐dose protocol may not defend the larger dose as compared to the low‐dose protocol combined with SharpView image enhancement.

The low‐dose images generally had a lower score than the full‐dose images, indicating that the low‐dose images required image enhancement to provide the same diagnostic performance as the full‐dose images. The SharpView enhanced low‐dose series were rated significantly higher than the low‐dose standard series. This may indicate that postprocessing filters like Sharpview could be a helpful tool in optimizing image quality.

All observers reported the highest mean score for the full‐dose series and lowest mean score for the low‐dose series. The mean score amongst the observers was most similar for the low‐dose SharpView enhanced series, and also the mean score was higher for SharpView enhanced series compared to the low‐dose series.

The interobserver differences were smaller for the low‐dose SharpView enhanced series than for both the full‐dose and the low‐dose series. Reader 2 had the highest score for the full‐dose series and lowest score for the low‐dose series compared to the other readers, but scored the low‐dose SharpView enhanced series equal to that of the others. This indicates a higher agreement between observers for the low‐dose SharpView enhanced protocol.

In a pilot multicenter study, the European guidelines on image quality criteria for CT were tested with regard to their utility in clinical practice for thoracic CT. The results from this study showed that the diagnostic criteria could be used to optimize CT procedures with respect to image quality and dose.^(^ ^20^ ^)^ Still, these criteria differ from normal diagnostic findings for a radiologist, and might seem unfamiliar to evaluate, and this might be the reason for the results of the ICC test where the score for the full‐dose series used in clinically practice in the hospital today were as low as 0.2. For some series, the dose levels recommended in these European guidelines are above the national reference doses for chest CT, and higher than the doses used in this study. Motion artifacts due to cardiac pulsation and breathing could also be factors influencing the observers' scores and the interobserver differences.

To utilize the full capacity of postprocessing strategies, identifying the maximum dose reduction without compromising the equal image quality would be required. Assessing image at several dose levels can identify this threshold dose. Multiple CT examinations would then be required, but would obviously not be acceptable from an ethical or radiation protection point of view. In the literature, low‐dose thoracic CT exams are performed as low as 30 mAs; this is the dose level used in our hospital. Especially for the mediastinal windows, the image quality may then in some cases be inadequate. To improve the image quality at this low dose level, SharpView postprocessing software may be one tool.

The image quality criteria chosen in this study should not be influenced by the fact that iodine contrast enhancement was utilized for the full‐dose series. The image appearance differed, however, and other pathology might be visualized better, and thus the diagnostic performance might have been improved. The low‐dose series and the low‐dose SharpView enhanced series are processed with the same raw data, and are thus identical except for the postprocessing.

For the evaluation of score for each individual patient and for each individual image quality criteria, the sample sizes were smaller than for the other results in the study. Therefore, these significance tests should be interpreted with care. A larger clinical trial should be performed.

One of the radiologists is a specialist in chest CT, while the other two are general radiologist. The results varied less between different dose levels and postprocessing for the thoracic radiologist compared to the others. This might indicate that the learning process of this study should have been expanded to reduce interobserver differences.

## V. CONCLUSIONS

In conclusion, using SharpView CT optimized with respect to anatomical region of interest and dose levels, gives a potential for improving diagnostic performance of low‐dose CT thoracic examinations.

## References

[1] de Berrington Gonzales A , Darby S . Risk of cancer from diagnostic X‐rays: estimates for the UK and 14 other countries. Lancet. 2004;363(9406):345–51. 15070562 10.1016/S0140-6736(04)15433-0

[2] Jessen KA . Going digital can help lower radiation dose. Diagnostic Imaging. 2004 Available from: http://www.diagnosticimaging.com/pacsweb/showArticle.jhtml?articleID=51202507

[3] Mettler FA , Wiest PW , Locken J , Kelsey C . CT scanning: patterns of use and dose. J Radiol Prot. 2000;20(4):353–59. 11140709 10.1088/0952-4746/20/4/301

[4] Børretzen I , Lysdahl KB , Olerud HM . [Radiology in Norway – examination frequency per 2002, trends in time, geographical variation and population dose.] Østerås, Norway: Norwegian Radiation Protection Authority; 2006.

[5] The 2007 Recommendations of the International Commission on Radiological Protection. ICRP Publication 103. Ann ICRP. 2007;37(2):1–332. 10.1016/j.icrp.2007.10.003 18082557

[6] Mayo JR , Aldrich J , Müller NL . Radiation exposure at chest CT: a statement of the Fleischner Society. Radiology. 2003;228(1):15–21. 12832569 10.1148/radiol.2281020874

[7] Martinsen ACT , Sæther HK , Olsen DR , Skaane P , Olerud HM . Reduction in dose from CT examinations of liver lesions with a new postprocessing filter: a ROC phantom study. Acta Radiol. 2008:49(3):303–09. 18365819 10.1080/02841850701793769

[8] Friberg EG , Widmark A , Olerud HM , Tynes T , Saxebøl G . [Guidance for use of medical X‐ray and MR equipment subjected to approval. Guidance to “Regulations for radiation protection and use of radiation”. Guidance No. 5.] In Norwegian. Østerås: Norwegian Radiation Protection Authority, 2005 Available from: http://www.nrpa.no/dav/bac3c61794.pdf

[9] Wormanns D , Ludwig K , Beyer F , Heindel W , Diederich S . Detection of pulmonary nodules at multirow‐detector CT: effectiveness of double reading to improve sensitivity at standard‐dose and low‐dose chest CT. Eur Radiol. 2005;15(1):14–22. 15526207 10.1007/s00330-004-2527-6

[10] Diederich S , Lenzen H , Windmann R , et al. Pulmonary nodules: experimental and clinical studies at low‐dose CT. Radiology. 1999;213(1):289–98. 10540674 10.1148/radiology.213.1.r99oc29289

[11] Karabulut N , Törü M , Gelebek V , Gülsün M , Ariyürek O M . Comparison of low‐dose and standard‐dose helical CT in the evaluation of pulmonary nodules. Eur Radiol. 2002;12(11):2764–69. 12386771 10.1007/s00330-002-1368-4

[12] Menezes RJ , Roberts HC , Paul NS , et al. Lung cancer screening using low‐dose computed tomography in at‐risk individuals: the Toronto experience. Lung Cancer. 2010;67(2):177–83. 19427055 10.1016/j.lungcan.2009.03.030

[13] Li X , Eshan S , DeLong D , et al. Paediatric MDCT: towards assessing the diagnostic influence of dose reduction on the detection of small lung nodules. Acta Radiol. 2009;16(7):872–80. 10.1016/j.acra.2009.01.028 19394875

[14] Pedersen JH , Ashraf H , Dirksen A , et al. The Danish randomized lung cancer CT screening trial – overall design and results of the prevalence round. J Thorac Oncol. 2009;4(5):608–14. 19357536 10.1097/JTO.0b013e3181a0d98f

[15] Linning E , Daqing M . Volumetric measurement pulmonary ground‐glass opacity nodules with multi‐detector CT: effect of various tube current on measurement accuracy – a chest CT phantom study. Acta Radiol. 2009;16(8):934–39. 10.1016/j.acra.2009.02.020 19409818

[16] Brenner DJ . Radiation risk potentially associated with low‐dose CT screening of adult smokers for lung cancer. Radiology. 2004;231(2):440–45. 15128988 10.1148/radiol.2312030880

[17] European Guidelines on Quality Criteria for Computed Tomography, EUR 16262. Available from http://www.drs.dk/guidelines/ct/quality/index.htm

[18] Granlund GH . In search of a general picture processing operator. Computer Graphics and Image Processing. 1978;8:155–73.

[19] Leander P , Söderberg M , Fält T , Gunnarasson M , Albertsson I . Post‐processing image filtration enabling dose reduction in standard abdominal CT. Radiat Prot Dosim. 2010. Pub. online March 5, 2010. 10.1093/rpd/ncq086 20207748

[20] Jurik A , Petersen J , Jessen KA , et al. Clinical use of image quality criteria in computed tomography: a pilot study. Radiat Prot Dosim. 2000;90(1–2):47–52.

